# Shear bond strength of a flash-free orthodontic adhesive system after thermal aging procedure

**DOI:** 10.4317/jced.55540

**Published:** 2019-02-01

**Authors:** Carlos González-Serrano, Eugenia Baena, María-Victoria Fuentes, Alberto Albaladejo, Manuel Míguez-Contreras, Manuel O. Lagravère, Laura Ceballos

**Affiliations:** 1 PhD Student, Area of Stomatology, Health Sciences Faculty, Rey Juan Carlos University, Alcorcón, Madrid, Spain; 2 Assistant Professor, Area of Stomatology, Health Sciences Faculty, Rey Juan Carlos University, Alcorcón, Madrid, Spain; 3 Associate Professor, Department of Surgery, Faculty of Medicine, University of Salamanca, Salamanca, Spain; 4 Assistant Professor, Department of Stomatology, Health Sciences Faculty, Alfonso X el Sabio, Madrid, Spain; 5 Associate Professor, School of Dentistry, Faculty of Medicine & Dentistry, University of Alberta, Edmonton, Canada; 6 Full Professor, Area of Stomatology, Health Sciences Faculty, Rey Juan Carlos University, Alcorcón, Madrid, Spain

## Abstract

**Background:**

The aim of this study was to compare the shear bond strength (SBS) of a flash-free and precoated orthodontic adhesive with a compomer orthodontic adhesive before and after thermocycling. The adhesive remnant index (ARI) was also determined for both adhesives.

**Material and Methods:**

The adhesive remnant index (ARI) was also determined for both adhesives. Material and Methods: A total of 120 human premolars were randomly divided into two groups (n=60) according to the orthodontic adhesive used: APC Flash-Free Adhesive Coated Appliance System (APC FF) or Transbond PLUS Color Change Adhesive (TP), as control. A SBS test was performed and ARI value for each specimen was also assessed. Results were analyzed by two-way ANOVA and Tukey’s Chi-square test (*p*<0.05).

**Results:**

SBS values were significantly influenced by thermocycling (*p*<0.01). Neither the orthodontic adhesive nor the interaction between adhesive and thermocycling statistically affected SBS results (*p*>0.05).

**Conclusions:**

APC FF and TP showed similar bond strength results. Thermocycling induced a significant decrease in SBS values for the two adhesives tested, without differences between 10,000 and 20,000 thermal cycles. Moreover, APC FF left less adhesive remnants on the enamel compared to TP.

** Key words:**APC Flash-Free, APC cement, aging, orthodontics, resin cements.

## Introduction

The use of adhesives and resin composites for bonding orthodontic brackets to etched teeth is considered a standard procedure in the orthodontic practice (1). Usually, the process of bonding brackets is performed by the manual application of an adhesive to the bracket base. However, in an attempt to perform easier and faster bonding procedures, light-cured adhesive pre-coated (APC) brackets (3M Unitek, Monrovia, CA, USA) were introduced in 1991.

From then onwards, different developments of APC systems were introduced in the market by manufacturer. 3M Unitek began by launching the APC Adhesive Coated Appliance System followed by the introduction of APC II Adhesive Coated Appliance System in 2000. Both of them were resin-based adhesives, but the latter was less viscous than its predecessor improving its handling properties and had a better blister package extending the expiration date and enhancing the preservation of the adhesive. The third development was introduced in 2002, which was the APC PLUS Adhesive Coated Appliance System (APC PLUS), which substantially modified the composition and properties of APC II Adhesive Coated Appliance System, being a compomer. Because of this, APC PLUS provides a higher tolerance to moisture compared to its predecessors as well as fluoride release during treatment. Furthermore, it has a characteristic pink color before polymerizing (after light curing the color fades away) to facilitate flash removal. In 2013, the APC Flash-Free (APC FF) technology was launched (APC Flash-Free Adhesive Coated Appliance System, 3M Unitek) with the intention of further decreasing the time needed for the bonding process, by eliminating the need of flash removal around the bracket once it is placed. More importantly, the transition from APC PLUS to APC FF also meant a modification in the composition of the adhesives, changing from a compomer to a low-viscosity resin. The APC FF orthodontic adhesive system is based on a nonwoven, polypropylene fiber material, which gets soaked by a low-viscosity resin. Once the bracket is placed, the compressible material lets the resin seep out, bonding the base of the bracket to the enamel (2). The difference between the chemical composition of a compomer and a low-viscocity resin makes the evaluation of this novel flash-free system necessary, since the achievement of a stable bond strength in the enamel-bracket interface remains a priority for the correct performance of the orthodontic treatment without an increase in bracket debonding rates (3). Moreover, the absence of flash around brackets when bonding, makes the evaluation of APC FF also interesting in order to decrease the formation of white spot lesion associated to adhesive excesses (4).

A limited amount of studies were found that evaluated APC FF (2,5-11). Three of these confirmed some of the benefits anticipated by the manufacturer, such as time savings during bracket bonding (7,8,11), even up to one third compared with a conventional orthodontic adhesive as has been recently stated by Grünheid *et al.* (11); or no need for flash removal after placement (7,9-11). Three of the manuscripts (6,8,9) evaluated the SBS obtaining high bond strength values. Those SBS tests were performed immediately after storing the samples in distilled water for 24 hours. However, no study has been found that analyzed the aging effect in the bond strength of the APC FF low-viscosity type resin as this a more realistic application to the orthodontic treatment time. Hence, in accordance with Lee *et al.* (6) a thermocycling study is warranted to elucidate the role of aging on the bonding properties of APC FF, as it is still unknown.

Since bonding efficacy (2,12), mechanical properties after aging (13) and type of failures after debonding (2) are crucial factors for orthodontists when selecting an orthodontic adhesive, the following study aimed to: (a) compare the SBS of the enamel-bracket interface between APC FF and a widely used conventional adhesive such as Transbond PLUS Color Change Adhesive (3M Unitek) (TP); (b) determine the influence of thermocycling on both adhesives; and (c) evaluate the amount of adhesive remnant on the tooth surface after bracket removal.

## Material and Methods

-*Specimen preparation*

One hundred and twenty sound human premolars extracted for orthodontic or periodontal reasons were stored in a 0.1% aqueous solution of thymol at 4ºC for no longer than 6 months. Their buccal enamel was sound with no damage due to the extraction process, and all the surfaces were cleaned from debris and soft tissue remnants before their storage.

Roots were submerged in a self-cured acrylic cylinder (Special Tray, Dentsply Sirona, Ballaigues, Switzerland) to allow stability for posterior SBS testing, according to previous studies (2,3,5,6). The conditioning protocol was applied following the manufacturer’s instructions. Firstly, the buccal crown surface of each premolar was brushed with fluoride-free pumice slurry for 15 seconds, and then rinsed and dried. Afterward, the enamel of the bonding surfaces was etched with 35% phosphoric acid (Transbond XT Etching Gel, 3M Unitek) for 30 seconds, rinsed for 30 seconds, and dried with oil-free and moisture-free air for 30 seconds. Then, a uniform coat of a light-cure adhesive primer (Transbond XT Primer, 3M Unitek) was applied.

The teeth were randomly divided into two groups according to the bonding agent applied to bond the ceramic brackets used in the study (Clarity Advanced, 3M Unitek). The application mode and chemical composition of the tested materials are reported in  Table 1.

**Table 1:**
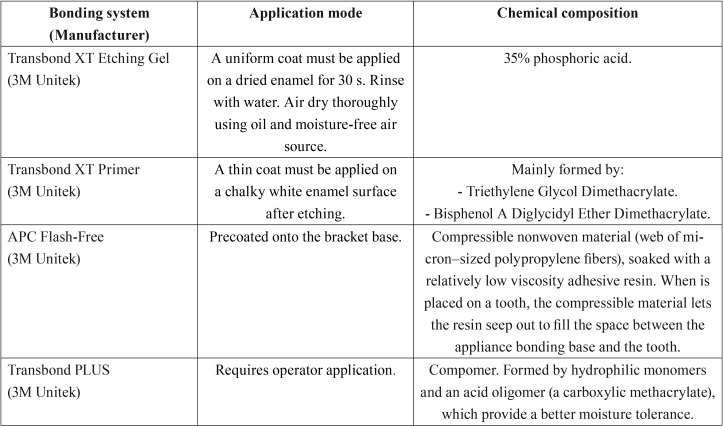
Application mode, chemical composition and characteristics of the conditioners and orthodontic adhesives evaluated, according to manufacturer’s instructions.

Group 1: A precoated bracket system was used, APC FF (3M Unitek). As the adhesive resin is already in the bracket base, brackets were placed immediately after primer application.

Group 2: The orthodontic adhesive TP (3M Unitek), that requires operator application, was placed onto the brackets base, and they were positioned on the buccal enamel surface. The adhesive TP was directly applied from the syringe to the bracket base.

All brackets were positioned and bonded by the same operator (C.G-S.) in order to standardize the bonding procedure. Once brackets were placed in the correct position (center of anatomic crown), they were pressed against the buccal surface of each premolar with the end of the bracket tweezers. For group 1, it was not necessary to remove the excesses around the bracket as APC FF does not generate flash. For group 2, flash was removed with the help of an explorer before polymerizing. A LED unit (Elipar S10, 3M ESPE, St Paul, MN, USA) was used to polymerize both bonding agents. Both orthodontic adhesives were polymerized for 40 seconds placing the end of the LED unit perpendicular to the bracket slot as recommended by the manufacturer, with an intensity of 1,200 mW/cm2.

Specimens from both groups were randomly divided into three subgroups (n = 20) according to the aging procedure carried out: storage in distilled water for 24 hours at 37ºC and thermocycled for 10,000 cycles or 20,000 cycles. Thermocycling was performed between 5ºC and 55ºC with a dwelling time of 30 seconds. The randomization was performed by assigning a 6-digit number from a random number table to each of the three subgroups, and then the premolars were randomly assigned to the previously mentioned subgroups.

According to Gale *et al.*, (14) 10,000 and 20,000 cycles thermocycling correspond to one and two years of clinical service respectively, which is the time usually required to complete an orthodontic treatment. Moreover, the temperatures and dwelling time applied have been recently confirmed by the Academy of Dental Materials (15) as the most suitable way of testing dental materials under thermal aging procedures.

-*Shear bond testing*

Afterwards, the acrylic cylinders were secured in a jig attached to the base plate of a universal testing machine (Instron 3345, Instron Corp., Canton, MA, USA). A chisel-edge plunger was mounted in the movable crosshead of the testing machine. It was positioned placing the leading edge aimed at the bracket body, between the base and the bracket wings, before it was brought into contact exerting a force parallel to a flat interface in the occlusal-apical direction. A crosshead speed of 0.5 mm/min was applied. The force required to dislodge the brackets was measured in Newtons (N), and the SBS was calculated in MegaPascals (MPa) by dividing the force values in N by the surface area of the bracket (mm2).

-*Type of failure and ARI*

Once brackets were debonded, enamel surfaces were analyzed by the primary investigator (C.G-S.) under a stereomicroscope (Olympus SZX7, Hamburg, Germany) at a magnification of 10X. The primary investigator previously received 20 hours of training on the use of the machine and how to obtain the measurements. To determine the type of failure, the ARI scores were determined according to the Årtun *et al.* (16) classification. They were categorized as: 0 = no adhesive remaining on the tooth in the bonding area; 1 = less than half of the adhesive remaining on the tooth; 2 = more than half of the adhesive remaining on the tooth; 3 = all adhesive remaining on the tooth with a distinct impression of the bracket mesh. In cases where the adhesive remaining on the enamel surface was around 50%, to determine whether it was an ARI 1 or 2, a greater magnification was used (25X) as well as the evaluation of the bracket base with the same magnifications (10X and 25X) was performed.

Finally, six specimens from each experimental subgroup were sputter-coated with gold (Bal-Tec Sputter Coater SCD 005, Witten, Germany) and observed under a scanning electron microscope (SEM) (Phillips XL30 ESEM, FEI Company, Hillsboro, OR, USA) to better analyze the type of failure of both adhesives.

-*Statistical analysis*

Normality and homogeneity were checked with Shapiro-Wilk and Levene tests, respectively. A two-way ANOVA was applied to analyze the influence of the orthodontic adhesive used and thermocycling procedure on SBS. Post-hoc comparisons were performed by Tukey’s test. Chi-square (χ2) test was performed to assess differences in the amount of adhesive remaining on the enamel surface after bracket debonding according to the orthodontic adhesive tested and the aging procedure.

All statistical tests were performed at a pre-set alpha of 0.05 using SPSS 22 for Windows software (IBM Corporation, Armonk, NY, USA).

## Results

 Table 2 shows the SBS values obtained for the two adhesives evaluated after different thermal aging procedures. Two-way ANOVA revealed that the SBS values were significantly influenced by the thermal aging procedure (*p* < 0.01). Neither the orthodontic adhesive nor the interaction between both factors (adhesive type-thermocycling) significantly affected SBS results (*p* > 0.05). Both adhesives showed similar SBS values either without thermocycling (24 hour water storage) or when thermocycling was performed for 10,000 and 20,000 cycles.

**Table 2:**
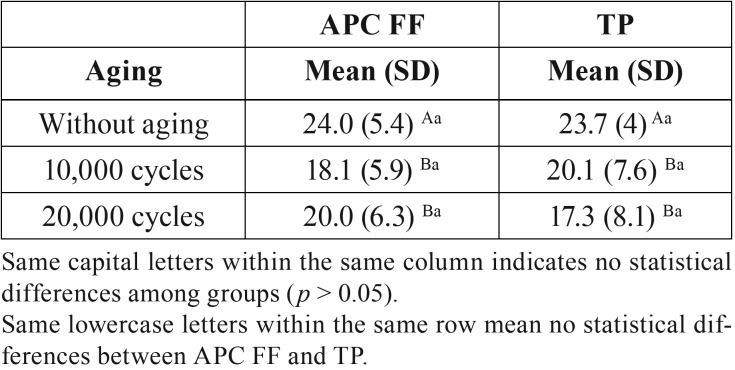
Mean SBS values (standard deviation) in MPa for the APC FF and TP groups after different thermal aging procedures (n = 20).

Thermocycling significantly decreased SBS values for the two adhesives tested. However, there was no significant difference in SBS mean values between 10,000 and 20,000 cycles for both groups.

The most prevalent type of failure for APC FF was at the enamel-adhesive level, with less than 50% of adhesive remaining on the enamel surface. Thermocycling did not alter this trend. On the other hand, with TP, bond failure occurred at the adhesive-bracket level, with all the adhesive remaining on the enamel surface in most cases; especially when thermocycling was performed.

**Figure 1:**
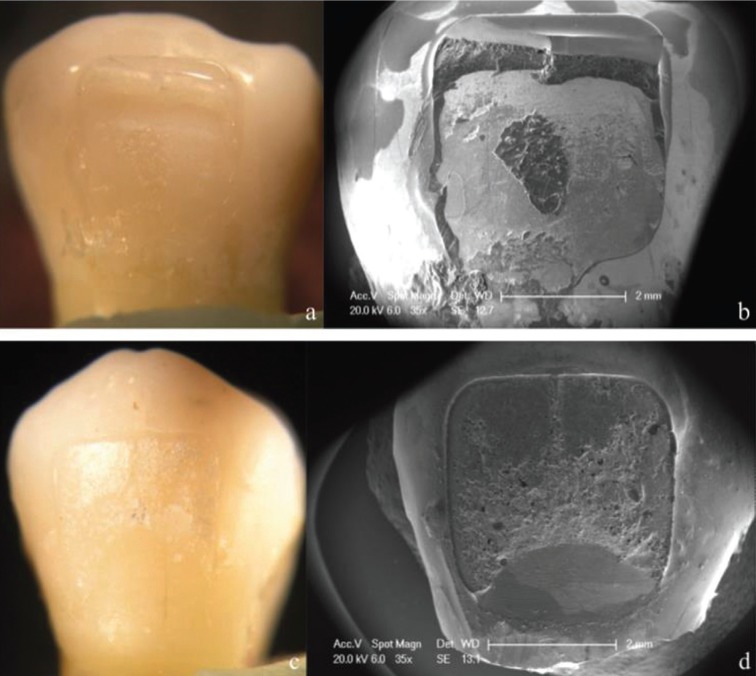
Stereromicroscope and SEM micrographs (35X) of representative failure modes for the experimental groups tested. a,b) show an ARI score 1 from APC FF without aging; c,d) show an ARI score 2 from TP without aging.

**Figure 2:**
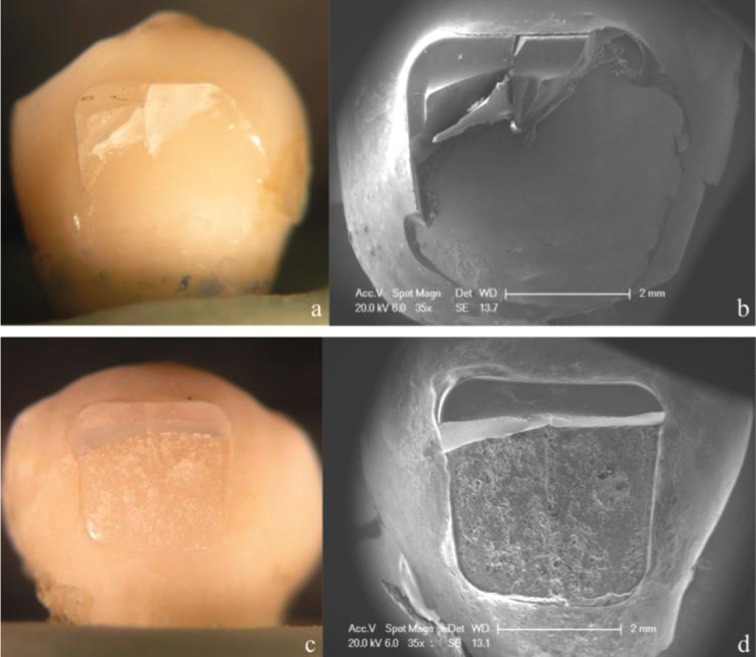
Stereromicroscope and SEM micrographs (35X) of representative failure modes for the experimental groups tested. a,b) show an ARI score 1 from APC FF with 10,000 cycles thermocycling; c,d) show an ARI score 3 from TP with 10,000 cycles thermocycling.

**Figure 3:**
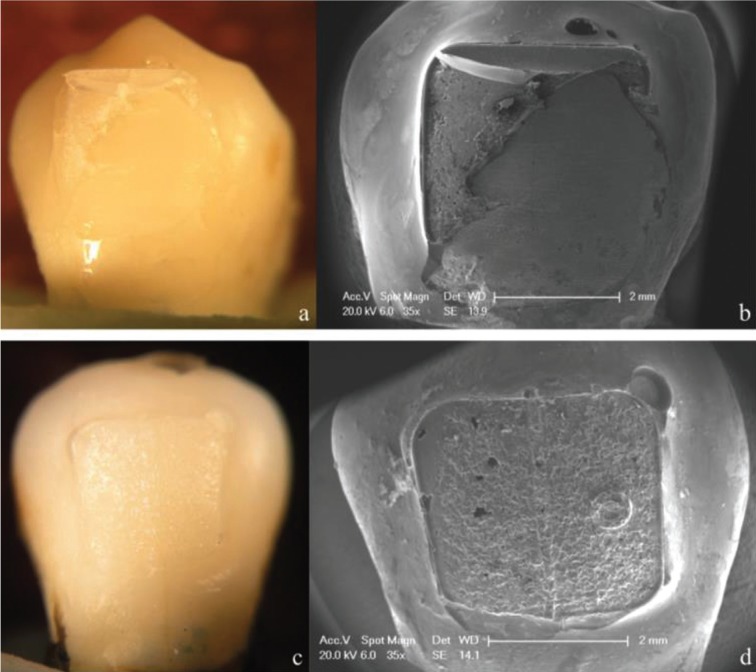
Stereromicroscope and SEM micrographs (35X) of representative failure modes for the experimental groups tested. a,b) show an ARI score 1 from APC FF with 20,000 cycles; c,d) show an ARI score 3 from TP with 20,000 cycles thermocycling.

Percentages of ARI scores after debonding are shown in  Table 3. According to Chi-square test results, there were statistically significant differences between the two adhesives tested (*p* < 0.001). Most of the brackets bonded with APC FF showed a score of ARI 1, regardless of the thermocycling procedure (Figs. 1a,b, 2a,b, 3a,b). However, thermocycling affected the failure mode when brackets were bonded with TP. Specimens not thermocycled obtained a score of ARI 2 in 65% of the cases (Fig. 1c-d). When thermocycling was performed for 10,000 cycles, ARI 2 and 3 were the most prevalent scores obtained (40% and 45% respectively) while for 20,000 cycles, ARI 3 was the most prevalent score (50%) (Figs. 2c,d, 3c,d).

**Table 3:**
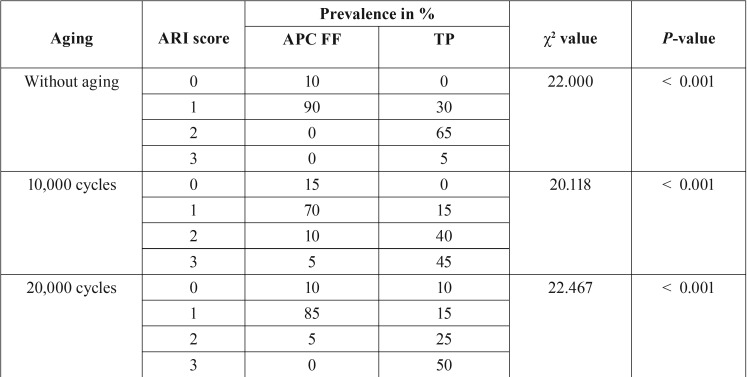
ARI scores obtained for each bonding system after debonding.

## Discussion

According to the results obtained, thermocycling influenced SBS values of both adhesives and no significant differences were found between APC FF and TP. Furthermore, significant differences concerning ARI scores between both adhesives were found. This reveals that a high bond to enamel can be achieved with both systems, as previously reported by other authors (6,8,10,11,17), even taking into consideration that each material has a different composition and is applied differently ( Table 1). TP has been widely studied, either in its pre-coated (APC PLUS) (18) or conventional (TP) presentation (17). In this study, TP has been used as the control adhesive since it is considered an optimum orthodontic adhesive with high SBS values (17).

The adhesion in the enamel-bracket interface ages through three different phenomena: mechanical, chemical and thermal changes. Although *in vitro* studies cannot exactly reproduce those conditions, they can, to some extent, simulate them through aging procedures (19). Thus, thermocycling is fully accepted and extensively used in experimental studies by the scientific community to reproduce the aging of dental materials produced by water and temperature changes in the oral cavity (14,15).

As noted, SBS values were significantly influenced by the thermal aging procedure. Several authors have described the deleterious effects of thermocycling in the adhesive interface after performing a SBS test (19-21). Hence, these number of cycles (10,000 and 20,000) are widely used in the scientific community when evaluating orthodontic adhesives (20,21). Mean SBS results obtained with both adhesives were higher than 6 to 8 MPa that are the values considered as clinically acceptable for orthodontic purposes (22). The present results were higher than those obtained by Lee *et al.* (6) for both adhesives. This could be explained by differences within the methodology as they used a 0.016-inch stainless steel wire to debond the brackets instead of a chisel. Thus, the force applied was in a gingivo-occlusal direction, which was the opposite direction to the one used for the present study. However, the mean SBS value obtained by Ansari *et al.* (8) and Marc *et al.* (9) for APC FF (20.13 MPa and 21.77 respectively), were very similar to the one obtained by us, being slightly lower. It is worth noting that although these studies were on SBS for APC FF, these studies did not evaluate thermocycling aging as the present study did. There is only one *in vivo* study that evaluated APC FF in an split-mouth randomized controlled clinical trial, although 2 manuscripts were published from it (10,11). After 1 year follow-up, they did not find any statistically significant difference between APC FF and a conventional orthodontic adhesive (APC II Adhesive Appliance System) with regard to bracket survival when ceramic brackets were used.

The continuous temperature changes performed in thermocycling have an influence on the bonding materials, causing the resin to expand and contract, as its thermal expansion coefficient is higher than that of the teeth; the higher the thermal expansion coefficient of a resin, the worse it would be for the adhesive interface as volumetric changes of the resin will be greater. Moreover, the presence of water during this procedure causes hygroscopic expansion as well as chemical degradation of the resinous components, a process known as plastification (23). This deleterious effect of thermal changes and hydrolytic degradation has been more relevant for the first 10,000 cycles, without a significant decrease in SBS values when the number of cycles was doubled; similarly to what happened to Turk *et al.* (21) who did not find any significant difference between 10,000 and 20,000 cycles for Transbond XT Light Cure Adhesive (3M Unitek).

Not only is the time used in the bonding procedure clinically relevant, but so is the time in debonding (5); therefore, the adhesive remaining on the tooth surface is also a key factor. When adhesive failure is produced at the enamel-adhesive interface, and is related to a lesser amount of adhesive on the tooth surface, there is a higher risk of enamel fracture (24), especially with ceramic brackets. Although at present APC FF is available with ceramic or metallic brackets, at the time of this study it was only available with ceramic brackets similar to other studies evaluating APC FF (2,5-8).

The ARI index is one of the most frequently used indexes in orthodontic adhesive testing (25). As detailed in  Table 3, ARI 1 was the most prevalent score obtained for APC FF independently of thermocycling. On the other hand, for TP, ARI 2 was the most prevalent score obtained for the non thermocycled group, whereas ARI 3 was the most prevalent score for 10,000 and 20,000 cycles of thermocycling. Lee *et al.* (6), similarly to what we found, recorded that all bracket failures in the APC FF group occurred within the adhesive (ARI 1). Concurrently, Foersch *et al.* (5) obtained similar results for the APC PLUS group (ARI 2,8 average score), whereas the values obtained for APC FF (ARI 2 average score) were slightly higher than those observed by us.

However, Grünheid *et al.* (2) found that in 94% of the brackets bonded with APC FF, all or most of the adhesive remained on the enamel after bracket removal (corresponding with ARI 2 and 3 values). This was different from the present study and could be explained by the methodology employed, since they used bovine incisors and a debonding instrument instead of a universal testing machine. Moreover, they used 2.5X magnification dental loupes, which could affect the ARI accurate scoring. Similarly to Grünheid *et al.* (2), Ansari *et al.* (8) obtained an ARI 3 score in 50% of the brackets bonded with APC FF, being the most prevalent score obtained for this adhesive, followed by ARI 2 (30%). Those differences with regard to our study could be explained due to the small sample used (n = 10) compared to 60 for each group in this study.

## Conclusions

Within the limitations of the present study, the following conclusions were obtained: 1) both adhesive systems, APC FF and TP, showed similar bond strength to enamel, 2) thermocycling significantly decreased the SBS values of both adhesives, without any significant difference between them, 3) APC FF left significantly less amount of adhesive on the tooth surface when debonding, presenting the failure at the enamel-adhesive interface, while TP presented it at the bracket-adhesive interface. Thermocycling did not affect this pattern for APC FF, but it did for TP.

## References

[1] Park SB, Son WS, Ko CC, Garcia-Godoy F, Park MG, Kim HI (2009). Influence of flowable resins on the shear bond strength of orthodontic brackets. Dent Mater J.

[2] Grünheid T, Sudit GN, Larson BE (2015). Debonding and adhesive remnant cleanup: an in vitro comparison of bond quality, adhesive remnant cleanup, and orthodontic acceptance of a flash-free product. Eur J Orthod.

[3] Öztürk F, Ersöz M, Öztürk SA, Hatunoğlu E, Malkoç S (2016). Micro-CT Evaluation of microleakage under orthodontic ceramic brackets bonded with different bonding techniques and adhesives. Eur J Orthod.

[4] Msallam FA, Grawish ME, Hafez AM, Abdelnaby YL (2017). Decalcification prevention around orthodontic brackets bonded to bleached enamel using different topical agents. Prog Orthod.

[5] Foersch M, Schuster C, Rahimi RK, Wehrbein H, Jacobs C (2016). A new flash-free orthodontic adhesive system: A first clinical and stereomicroscopic study. Angle Orthod.

[6] Lee M, Kanavakis G (2016). Comparison of shear bond strength and bonding time of a novel flash-free bonding system. Angle Orthod.

[7] Kim J, Kanavakis G, Finkelman MD, Lee M (2016). Microleakage under ceramic flash-free orthodontic brackets after thermal cycling. Angle Orthod.

[8] Ansari MY, Agarwal DK, Gupta A, Bhattacharya P, Ansar J, Bhandari R (2016). Shear bond strength of ceramic brackets with different base designs: Comparative in-vitro study. J Clin Diagn Res.

[9] Marc MG, Bazert C, Attal JP (2018). Bond strength of pre-coated flash-free adhesive ceramic brackets. An in vitro comparative study on the second mandibular premolars. Int Orthod.

[10] Grünheid T, Larson BE A comparative assessment of bracket survival and adhesive removal time using flash-free or conventional adhesive for orthodontic bracket bonding: A split-mouth randomized controlled clinical trial. Angle Orthod.

[11] Grünheid T, Larson BE (2018). Comparative assessment of bonding time and 1-year bracket survival using flash-free and conventional adhesives for orthodontic bracket bonding: A split-mouth randomized controlled clinical trial. Am J Orthod Dentofacial Orthop.

[12] Barbosa IV, Ladewig VM, Almeida-Pedrin RR, Cardoso MA, Santiago Junior JF, Conti ACCF (2018). The association between patient's compliance and age with the bonding failure of orthodontic brackets: a cross-sectional study. Prog Orthod.

[13] Elekdag-Turk S, Turk T, Isci D, Ozkalayci N (2008). Thermocycling effects on shear bond strength of a self-etching primer. Angle Orthod.

[14] Gale MS, Darvell BW (1999). Thermal cycling procedures for laboratory testing of dental restorations. J Dent.

[15] Armstrong S, Breschi L, Özcan M, Pfefferkorn F, Ferrari M, Van Meerbeek B (2017). Academy of Dental Materials guidance on in vitro testing of dental composite bonding effectiveness to dentin/enamel using micro-tensile bond strength (µTBS) approach. Dent Mater.

[16] Årtun J, Bergland S (1984). Clinical trials with crystal growth conditioning as an alternative to acid-etch enamel pretreatment. Am J Orthod.

[17] Goswami A, Mitali B, Roy B (2014). Shear bond strength comparison of moisture-insensitive primer and self-etching primer. J Orthod Sci.

[18] Vicente A, Bravo LA (2007). Shear bond strength of precoated and uncoated brackets using a self-etching primer. Angle Orthod.

[19] Morresi AL, D'Amario M, Capogreco M, Gatto R, Marzo G, D'Arcangelo C (2014). Thermal cycling for restorative materials: does a standardized protocol exist in laboratory testing? A literature review. J Mech Behav Biomed Mater.

[20] Sokucu O, Siso SH, Ozturk F, Nalcaci R (2010). Shear bond strength of orthodontic brackets cured with different light sources under thermocycling. Eur J Dent.

[21] Turk T, Elekdag-Turk S, Isci D, Cakmak F, Ozkalayci N (2010). Shear bond strength of a self-etching primer after 10,000 and 20,000 thermal cycles. J Adhes Dent.

[22] Reynolds IR (1975). A review of direct orthodontic bonding. Br J Orthod.

[23] Mohammadi E, Pishevar L, Mirzakouchaki Boroujeni P (2017). Effect of food simulating liquids on the flexural strength of a methacrylate and silorane-based composite. PLoS One [Internet].

[24] Katona TR (1997). Stresses developed during clinical debonding of stainless steel orthodontic brackets. Angle Orthod.

[25] Montasser MA, Drummond JL (2009). Reliability of the adhesive remnant index score system with different magnifications. Angle Orthod.

